# Reply to ‘Anisotropy governs strain stiffening in nanotwinned-materials’

**DOI:** 10.1038/s41467-018-03967-6

**Published:** 2018-04-23

**Authors:** Bing Li, Hong Sun, Changfeng Chen

**Affiliations:** 1 School of Physics and Astronomy and Key Laboratory of Artificial Structures and Quantum Control (Ministry of Education), Shanghai Jiao Tong University, 200240 Shanghai, China; 2 Department of Physics and High Pressure Science and Engineering Center, University of Nevada, Las Vegas, NV 89154 USA

In their correspondence, Taheri Mousavi et al. presented molecular dynamics (MD) calculation results of nano-twined Cu (nt-Cu) with twinning spacing below 3 nm under Vickers indentation shear deformation in the (001)[110] direction of the nt-Cu supercell, which showed the same strain-stiffening effect similar to what was reported for nano-twined BN (nt-cBN) and nano-twined diamond (nt-Dia) in our previous papers^1, 2^. They argued that as the early experiments^3^ reported nt-Cu softening under tensile deformation below a critical twinning spacing (*λ* < 15 nm), their finding places significant doubt on the validity of our model in explaining the observed Vickers hardness enhancement of nt-cBN and nt-Dia^4, 5^, where the twinning spacing is about 4– 5 nm. First of all, the strain-stiffening effect found by first principles and MD calculations for nt-cBN, nt-Dia in our work^1, 2^ and nt-Cu in the correspondence comes from the transformation of atomic bonding on twin boundaries from easy sliding configurations to hard sliding configurations under indentation shear deformation, and such strain-stiffening mechanism does not exist in tensile deformation, as we showed in our works^1, 2^. The comparison of the calculated strain-stiffening effect of nt-Cu under indentation shear deformation with its experimental tensile strength made in the correspondence is not meaningful. Second, the authors of the correspondence seem to be not aware of the recent experimental report, where unprecedented strengthening of the tensile strength up to 3 GPa was observed experimentally for nano-twined metal Ni_83.6_Mo_14_W_2.4_ with twinning spacing at *λ* = 1.8 nm^6^, which has a nano-twinning enhanced tensile strength more than two times of those of nano-crystalline Ni and nano-twined Cu. These recent findings^6^ suggest that even the effects of nano-twinning on the tensile strengths of nano-twining metals depend critically on the sample qualities, structures, material compositions, and experimental processes. Third, the strain-stiffening effect we found in nt-cBN (or nt-Dia)^1, 2^ is different from the Hall-Petch effect, with the former describing the intrinsic indentation shear strength of ideal nt-cBN (or nt-Dia) without defects and dislocations, while the later describing the blocking of dislocation motions by nano-twin or other grain boundaries. In addition to the Hall-Petch effect, our results show that nano-twining in nt-cBN (or nt-Dia) can flip the easy atomic bonding to hard atomic bonding across the twining interfaces under indentation, which suppresses the original easy shear mode in single-crystal cBN (or diamond) and enhances the ideal indentation strength of nt-cBN (or nt-Dia) to twice that of single-crystal cBN (or diamond)^1, 2^, consistent with the experimental results^4, 5^. It should be noted, however, the measured hardness of real nano-twining materials, including nt-cBN, nt-Dia or nt-Cu, also depends on other qualities, such as defects, dislocations, other grain boundaries, different mechanisms of dislocation motions, etc. of the samples.
